# Fatting the brain: a brief of recent research

**DOI:** 10.3389/fncel.2013.00144

**Published:** 2013-09-09

**Authors:** Ghulam Hussain, Florent Schmitt, Jean-Philippe Loeffler, Jose-Luis Gonzalez de Aguilar

**Affiliations:** ^1^UMR_S 1118, Université de StrasbourgStrasbourg, France; ^2^Mécanismes Centraux et Périphériques de la Neurodégénérescence, U1118, Institut National de la Santé et de la Recherche Médicale, Faculté de Médecine, Université de StrasbourgStrasbourg, France

**Keywords:** monounsaturated fatty acid, neurodegenerative disease, neurological disease, peripheral nerve, saturated fatty acid, polyunsaturated fatty acid

## Abstract

Fatty acids are of paramount importance to all cells, since they provide energy, function as signaling molecules, and sustain structural integrity of cellular membranes. In the nervous system, where fatty acids are found in huge amounts, they participate in its development and maintenance throughout life. Growing evidence strongly indicates that fatty acids in their own right are also implicated in pathological conditions, including neurodegenerative diseases, mental disorders, stroke, and trauma. In this review, we focus on recent studies that demonstrate the relationships between fatty acids and function and dysfunction of the nervous system. Fatty acids stimulate gene expression and neuronal activity, boost synaptogenesis and neurogenesis, and prevent neuroinflammation and apoptosis. By doing so, they promote brain development, ameliorate cognitive functions, serve as anti-depressants and anti-convulsants, bestow protection against traumatic insults, and enhance repairing processes. On the other hand, unbalance between different fatty acid families or excess of some of them generate deleterious side effects, which limit the translatability of successful results in experimental settings into effective therapeutic strategies for humans. Despite these constraints, there exists realistic evidence to consider that nutritional therapies based on fatty acids can be of benefit to several currently incurable nervous system diseases.

## INTRODUCTION

Fatty acids represent a class of lipids that are crucial components of all mammalian cells. They display a variety of biological functions to maintain vital cellular processes at various levels. Thus, fatty acids provide energy, function as signaling molecules, and sustain structural integrity of cellular membranes. They are of particular importance for the nervous system for two major reasons. First, the nervous system possesses a very high concentration of fatty acids, second only to adipose tissue (Etschmaier et al., 2011). Second, these fatty acids participate actively both in the development of the nervous system during embryonic and early postnatal life, and in its maintenance throughout adulthood and natural aging (Uauy and Dangour, 2006; Rombaldi Bernardi et al., 2012). Along with these actions, currently incurable pathological conditions of the nervous system, including neurodegenerative diseases, mental disorders, stroke, and trauma, involve deregulated contents of fatty acids. It is therefore believed that these changes contribute in their own right by as yet incompletely understood mechanisms to those pathological processes. In consequence, the roles of fatty acids in health and disease of the nervous system have been intensively investigated in the last few decades. In this piece of work, we focus mainly on studies published during the last five years to show the diversity in the fatty acids implicated in function and dysfunction of the nervous system. The detailed mechanisms of action of fatty acids at the molecular level are not treated in this article, since they are the subject of other recently published reviews (Georgiadi and Kersten, 2012; Yamashima, 2012).

## SOME ASPECTS OF THE BIOCHEMISTRY OF FATTY ACIDS

**FIGURE 1:**
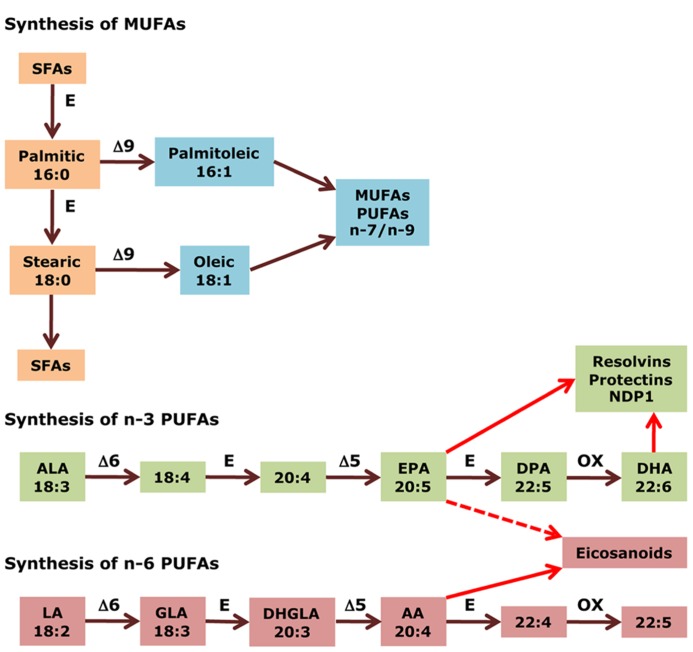
**Biosynthesis of fatty acids.** Medium- to long chain SFAs are successively transformed by the action of elongases (E) into palmitic acid (16:0), which is then either elongated to stearic acid (18:0), and other long chain SFAs, or desaturated, together with stearic acid (18:0), by δ9 desaturase to produce *de novo* MUFAs of the n-7 and n-9 series, such as palmitoleic acid (16:1) and oleic acid (18:1). In the case of PUFAs, δ6 and δ5 desaturases work cooperatively with elongases to introduce double bonds and extend the aliphatic chain in a successive manner, from ALA (18:3 n-3) to EPA (20:5 n-3) in the n-3 series, and from LA (18:2 n-6) to AA (20:4 n-6) in the n-6 series. Afterward, these end products are further elongated, desaturated, and submitted to peroxisomal β-oxidation (all three steps indicated by OX) to yield DHA (22:6 n-3) and docosapentaenoic acid (22:5 n-6), respectively. Finally, AA (20:4 n-6) is the precursor of potent pro-inflammatory eicosanoids. EPA (20:5 n-3) produces less potent (dashed arrow) eicosanoids and, together with DHA (22:6 n-3), gives rise to docosanoids with anti-inflammatory properties (i.e., resolvins and protectins). GLA, γ-linolenic acid; DHGLA, dihomo-γ-linolenic acid.

According to the IUPAC definition, fatty acids are “aliphatic monocarboxylic acids derived from or contained in esterified form in an animal or vegetable fat, oil or wax” (IUPAC, 1997). Naturally occurring fatty acids mostly consist of an unbranched 4–28 carbon chain that is usually composed of an even number of carbon atoms. On the basis of the carbon chain length, fatty acids are classified into short- (less than six carbon atoms), medium- (6–12 carbon atoms), long- (14–22 carbon atoms), and very long chain fatty acids (more than 22 carbon atoms). Fatty acids in which the aliphatic chain is fully composed of single bonds between carbon atoms are named as saturated fatty acids (SFAs), whereas fatty acids with one or more than one carbon–carbon double bond are called unsaturated fatty acids. Based on the number of double bonds, unsaturated fatty acids are further divided into mono-unsaturated fatty acids (MUFAs) and polyunsaturated fatty acids (PUFAs; **Table 1**). Long chain SFAs have relatively high melting points that make them to appear solid at room temperature. Therefore, the body possesses a mechanism to introduce double bonds in the carbon chain, which lowers the melting point and permits functioning in a physiological environment. There are four fatty acid desaturases documented in humans that selectively catalyze the introduction of a double bond in different positions of the carbon chain. Δ-9 desaturase, also known as stearoyl-CoA desaturase (SCD), is charged with synthesizing MUFAs, mainly palmitoleic acid (16:1) and oleic acid (18:1), by introducing a double bond between carbon atoms nine and 10 from the carboxylic acid end (**Figure 1**; Enoch et al., 1976). Δ-4, Δ-5, and Δ-6 desaturases introduce a double bond at carbon positions 4, 5, and 6, respectively, and work cooperatively with elongases, which are responsible for the extension of the aliphatic chain. The combined actions of desaturases and elongases are implicated in the synthesis of PUFAs (Nakamura and Nara, 2004).

**Table 1:** Most typical fatty acids.

Systematic name	Common name	Abbreviation	C:D n-X^1^
**Saturated fatty acids (SFAs)**
Butanoic	Butyric		4:0
Hexanoic	Caproic		6:0
Octanoic	Caprylic		8:0
Decanoic	Capric		10:0
Dodecanoic	Lauric		12:0
Tetradecanoic	Myristic		14:0
Hexadecanoic	Palmitic		16:0
Octadecanoic	Stearic		18:0
Eicosanoic	Arachidic		20:0
Docosanoic	Behenic		22:0
Tetracosanoic	Lignoceric		24:0
**Mono-unsaturated fatty acids (MUFAs)**
*cis*-9-Hexadecenoic	Palmitoleic		16:1
*cis*-9-Octadecenoic	Oleic		18:1
*cis*-13-Docosenoic	Erucic		22:1
*cis*-15-Tetracosenoic	Nervonic		24:1
**Polyunsaturated fatty acids (PUFAs)**
*cis*-9,*cis*-12-*cis*-15-Octadecatrienoic	α-Linolenic	ALA	18:3 n-3
*cis*-6,*cis*-9,*cis*-12,*cis*-15-Octadecatetraenoic	Stearidonic	SDA	18:4 n-3
*cis*-8,*cis*-11,*cis*-14,*cis*-17-Eicosatetraenoic	Eicosatetraenoic	ETE	20:4 n-3
*cis*-5, *cis*-8,*cis*-11,*cis*-14,*cis*-17-Eicosapentaenoic	Eicosapentaenoic	EPA	20:5 n-3
*cis*-7,*cis*-10,*cis*-13,*cis*-16, *cis*-19-Docosapentaenoic	Docosapentaenoic	DPA	22:5 n-3
*cis*-4,*cis*-7,*cis*-10,*cis*-13,*cis*-16,*cis*-19-Docosahexaenoic	Docosahexaenoic	DHA	22:6 n-3
*cis*-9,*cis*-12-Octadecadienoic	Linoleic	LA	18:2 n-6
*cis*-6,*cis*-9,*cis*-12-Octadecatrienoic	γ-Linolenic	GLA	18:3 n-6
*cis*-8,*cis*-11,*cis*-14-Eicosatrienoic	Dihomo-γ-linolenic	DHGLA	20:3 n-6
*cis*-5,*cis*-8,*cis*-11,*cis*-14-Eicosatetraenoic	Arachidonic	AA	20:4 n-6
*cis*-7,*cis*-10,*cis*-13,*cis*-16-Docosatetrtaenoic	Docosatetraenoic		22:4 n-6
*cis*-4,*cis*-7,*cis*-10,*cis*-13,*cis*-16-Docosapentaenoic	Docosapentaenoic		22:5 n-6

1 This nomenclature designates the number of carbon atoms in the fatty acid (C), the number of double bonds (D), and the position of the first double bond counting from the terminal methyl carbon (n-X).

According to the position of the first double bond from the methyl end of the fatty acid chain, the most important PUFAs for humans can be divided into two families: n-6 and n-3 PUFAs. Linoleic acid (LA, 18:2 n-6) is the parent fatty acid of n-6 PUFAs, which produces principally arachidonic acid (AA, 20:4 n-6), whereas α-linolenic acid (ALA, 18:3 n-3) is the parent fatty acid of n-3 PUFAs, which gives rise mainly to eicosapentaenoic acid (EPA, 20:5 n-3) and subsequently docosahexaenoic acid (DHA, 22:6 n-3; **Figure 1**). Both LA (18:2 n-6) and ALA (18:3 n-3) cannot be synthesized indigenously by the human body, so that they must be supplied with food, and such fatty acids are termed as essential fatty acids (Ruzickova et al., 2004; Lands, 2012). In spite of the fact that the body is able to metabolize these essential fatty acids, the efficiency of conversion is low. Hence, the availability not only of essential precursors but also of some of their metabolites, such as EPA (20:5 n-3) and DHA (22:6 n-3), greatly depends on dietary support (Brenna et al., 2009). Alternatively, PUFAs can also be made available by enzymatic processing of membrane phospholipids by phospholipases (Lee et al., 2011). Whatever pathway is involved, several PUFAs can be metabolized by cyclo-oxygenases, lipo-oxygenases, or cytochrome P450 mono-oxygenases to produce other compounds with important biological functions. AA (20:4 n-6) and, to a lesser extent, EPA (20:5 n-3) are transformed into potent pro-inflammatory eicosanoids. Additionally, EPA (20:5 n-3) and DHA (22:6 n-3) generate opposing anti-inflammatory docosanoids, including resolvins and protectins such as neuroprotectin-D1 (NPD1; Bazan, 2009; **Figure 1**).

## EVIDENCE OF THE IMPORTANCE OF FATTY ACIDS FOR HEALTH AND DISEASE OF THE NERVOUS SYSTEM

### FATTY ACIDS AND BRAIN DEVELOPMENT

Mother’s own resources, via placenta and milk, provide most of the n-3 PUFAs necessary for brain development during fetal and early postnatal life. Due to this high demand of the developing nervous system in the progeny, maternal brain levels of DHA (22:6 n-3) exhaust during pregnancy and lactation period (Chen and Su, 2012). Thus, enhanced provision or adequate supply of n-3 PUFAs at these stages can yield positive effects on offspring brain development. For instance, increased expression of neuron specific enolase, glial fibrillary acidic protein, and myelin basic protein was observed in pups from mice fed on n-3 PUFA enriched diet, administered from two months prior to mouse conception to end of lactation period (Tian et al., 2011). Similarly, postnatal supplementation of ALA (18:3 n-3), the parent precursor of n-3 PUFAs, enhanced cell proliferation and early neuronal differentiation, while its deprivation resulted in increased proportion of apoptosis in the dentate gyrus of unweaned pups. This ameliorating effect was offset by maternal ALA (18:3 n-3) deficiency during gestation period, suggesting that ALA (18:3 n-3) is not only required at postnatal stages but is also essential for fetal brain development (Niculescu et al., 2011). Importantly, such diets given at perinatal stages may have long lasting consequences in the adulthood. Thus, the abundance of n-3 PUFAs in the diet of pregnant females revealed essential for the development of the glutamatergic system and normal behavior performance in the adult offspring (Moreira et al., 2010a). Also, motor coordination was ameliorated in adulthood when rats were fed on DHA (22:6 n-3) and EPA (20:5 n-3) supplementation starting from gestation stage to postnatal age of 90 days (Coluccia et al., 2009). Finally, n-3 PUFA enriched diets also improved reference and working memory in offspring rats when supplied to mother at gestation stage (Chung et al., 2008).

Frequently, the impact of dietary fatty acids depends on a balance between different types. In a study to assess the effects of quality and quantity of several high fat diets, mice were nourished with various concentrations and types of fats mingled with normal chow. It was noticed that these diets not only modified the lipid profile in brain but also altered spatial memory and learning ability of the pups in a different manner (Yu et al., 2010). In another study, when mice were fed on diets supplemented with either SFAs or MUFAs, MUFAs promoted insulin sensitivity and cortical activity while SFAs did not (Sartorius et al., 2012). Lastly, it is noteworthy that the intake of sufficient quantity of MUFAs prevented the age related deletion of mitochondrial DNA in the brain of aged animals (Ochoa et al., 2011).

### FATTY ACIDS AND NEURODEGENERATIVE DISORDERS

The altered amounts of different classes of fatty acids in the nervous system may contribute positively or negatively to any given neuropathological process (**Table 2**). Using APP-C99-transfected COS-7 cells, a cellular model of Alzheimer’s disease-like degeneration, a study was carried out to investigate the class of fatty acids that was thought to influence the production of Aβ peptide, which is a major neuropathological hallmark of disease. It was shown that palmitic acid (16:0), stearic acid (18:0), upstream n-3 PUFAs, and AA (20:4 n-6) triggered higher secretion of Aβ peptide compared to long chain downstream n-3 PUFAs and MUFAs (Amtul et al., 2011a). These findings were corroborated *in vivo* by using a transgenic mouse model of early-onset Alzheimer’s disease that expresses the double-mutant form of human APP, which is the precursor protein responsible for the synthesis of Aβ peptide. Decreased levels of Aβ peptide and less accumulation in the form of amyloid plaques were observed in the brain of mice nourished with a diet enriched in n-3 PUFAs, mainly DHA (22:6 n-3; Amtul et al., 2011a). Not only extraneously supplied but endogenously synthesized n-3 PUFAs can suppress the synthesis of Aβ peptide and the formation of amyloid plaques. Lebbadi et al. (2011) crossed 3xTg-AD mice, a model of Alzheimer’s disease, with transgenic mice expressing Δ-3 desaturase (Fat-1) from *Caenorhabditis elegans*, which endogenously converts n-6 PUFAs into n-3 PUFAs. It was observed that the double transgenic 3xTg-AD/Fat-1 mice had increased brain levels of DHA (22:6 n-3) and lower levels of Aβ peptide. Similarly, MUFAs, mainly oleic acid (18:1 n-9), were also shown to inhibit the production of Aβ peptide and amyloid plaques both *in vitro* and *in vivo* (Amtul et al., 2011b). In contrast, n-6 PUFAs, such as AA (20:4 n-6), aggravated Alzheimer’s disease neuropathology, by increasing the synthesis of Aβ peptide (Amtul et al., 2012).

**Table 2:** Changes in brain fatty acid composition in pathological conditions.

Fatty acid	Disease	Tendency	Reference^1^
**Saturated fatty acids (SFAs)**
Myristic (14:0)	Depression	Down	Conklin et al. (2010)
Palmitic (16:0)	Alzheimer	Up	Fraser et al. (2010)
Palmitic (16:0)	Parkinson	Up	Fabelo et al. (2011)
Palmitic (16:0)	Depression	Down	Conklin et al. (2010)
Palmitic (16:0)	Depression	Up	Hamazaki et al. (2012)
Stearic (18:0)	Alzheimer	Down	Fraser et al. (2010)
Stearic (18:0)	Parkinson	Up	Fabelo et al. (2011)
Stearic (18:0)	Depression	Down	Conklin et al. (2010)
**Mono-unsaturated fatty acids (MUFAs)**
Palmitoleic (16:1)	Alzheimer	Up	Astarita et al. (2011)
Palmitoleic (16:1)	Depression	Down	Conklin et al. (2010)
Oleic (18:1)	Alzheimer	Down	Mart ïn et al. (2010)
Oleic (18:1)	Alzheimer	Up	Fraser et al. (2010)
Oleic (18:1)	Alzheimer	Up	Astarita et al. (2011)
Oleic (18:1)	Depression	Up	Hamazaki et al. (2012)
Erucic (22:1)	Alzheimer	Up	Astarita et al. (2011)
Nervonic (24:1)	Alzheimer	Up	Astarita et al. (2011)
**Polyunsaturated fatty acids (PUFAs)**
EPA (20:5 n-3)	Depression	Down	Lin et al. (2010)
DPA (22:5 n-3)	Depression	Down	Conklin et al. (2010)
DHA (22:6 n-3)	Alzheimer	Down	Mart ïn et al. (2010)
DHA (22:6 n-3)	Parkinson	Down	Fabelo et al. (2011)
DHA (22:6 n-3)	Depression	Down	Conklin et al. (2010)
DHA (22:6 n-3)	Depression	Down	Lin et al. (2010)
LA (18:2 n-6)	Depression	Down	Conklin et al. (2010)
AA (20:4 n-6)	Parkinson	Down	Fabelo et al. (2011)
AA (20:4 n-6)	Depression	Down	Conklin et al. (2010)
Docosatetraenoic (22:4 n-6)	Depression	Down	Conklin et al. (2010)
Docosatetraenoic (22:4 n-6)	Schizophrenia	Down	Hamazaki et al. (2012)

1 This table summarizes recent studies cited in the text. It is concluded that, whatever disease condition is considered, PUFA levels were systematically decreased, whereas MUFA amounts often appeared increased. In contrast, there was no clear-cut tendency in the changes of the proportions of SFAs.

The results obtained in experimental models of Alzheimer’s disease have been confirmed, at a certain extent, by studies performed on human brain. Thus, decreased levels of PUFAs and MUFAs, particularly DHA (22:6 n-3) and oleic acid (18:1 n-9), respectively, were observed in the brain of Alzheimer’s disease patients (Martïn et al., 2010). However, other studies reported that, although the abundance of DHA (22:6 n-3) varied highly among patients, the mean quantity of this PUFA did not differ significantly when compared to healthy brains (Fraser et al., 2010). This study also showed that levels of stearic acid (18:0) were reduced remarkably in frontal and temporal cortex, while those of oleic acid (18:1 n-9) were increased in both parts; also, levels of palmitic acid (16:0) appeared increased in the parietal cortex (Fraser et al., 2010). These *a priori* puzzling abnormalities in MUFAs could be a result of alterations in the expression of MUFA synthesizing genes. Thus, levels of MUFAs in hippocampus, frontal cortex and temporal cortex were elevated in Alzheimer’s disease patients, as was the expression of the SCD isomers SCD1, SCD5a, and SCD5b. In addition, the ratio of MUFAs to SFAs, an index of desaturase activity, was reported to be negatively correlated with the degree of cognitive performance (Astarita et al., 2011).

Less is known about the changes of fatty acids in other neurodegenerative conditions. Fabelo et al. (2011) reported that lipid rafts from brain cortices of patients with Parkinson disease displayed significantly decreased levels of n-3 and n-6 PUFAs, particularly DHA (22:6 n-3) and AA (20:4 n-6), respectively, while SFAs, mainly palmitic acid (16:0) and stearic acid (18:0), were noted augmented, as compared to control subjects. In another study, the effects of diets rich in n-3 or n-6 PUFAs were assessed on cuprizone-induced experimental demyelination, an animal model for multiple sclerosis. It was observed that n-3 PUFAs from various sources affected the pathological phenotype differently; for example, a diet containing n-3 PUFAs from salmon ameliorated the behavioral deficits induced by cuprizone, whereas a diet containing n-3 PUFAs from cod affected similarly as n-6 PUFA enriched or control diet did, suggesting that not only the type of PUFA but its origin is also to consider when prescribing a diet based remedy (Torkildsen et al., 2009). Contrasting these findings, other studies did not corroborate the protective effects of n-3 PUFAs against multiple sclerosis and concluded that neither n-3 nor n-6 PUFAs had any effect on disease progression or remedial influence (Wergeland et al., 2012). Moreover, dietary administration of EPA (20:5 n-3) even accelerated disease progression in mice expressing a mutated form of Cu/Zn-superoxide dismutase (SOD1), which is a model of neuromuscular degeneration as caused by amyotrophic lateral sclerosis (Yip et al., 2013).

### FATTY ACIDS AND TRAUMATIC INJURY TO THE NERVOUS SYSTEM

Several recent studies have provided evidence that n-3 PUFAs can exert protection against neuronal injury triggered by hypoxia or ischemia. In neonates, these fatty acids protected neurons following hypoxia/ischemia by modulating the microglial inflammatory response through inhibition of the nuclear factor-κB (NF-κB) dependent pathway (Zhang et al., 2010). However, it is important to mention that consistent increased intake of n-3 PUFAs can also affect adversely in some cases. In this respect, a diet rich in EPA (20:5 n-3) and DHA (22:6 n-3) enhanced the risk for intracerebral hemorrhagic stroke in rats, and caused oxidative damage to the brain, probably due to the fact that a high PUFA content increased the danger of lipid peroxidation. Alternatively, n-3 PUFA intake was reported to affect blood viscosity, vasoconstriction, platelet aggregation, and blood clotting ultimately leading to hemorrhaging (Park et al., 2009).

There is also evidence that certain fatty acids have the potential to improve the recovery of the injured spinal cord. Hirakawa et al. (2010) reported that trans-2-decenoic acid ethyl ester, a medium-chain fatty acid derivative, increased the expression of extracellular signal-regulated protein kinases 1 and 2 (ERK1/2) in cultured cortical neurons and at the site of injury in a rat spinal cord injury model. Indeed, the administration of trans-2-decenoic acid ethyl ester ameliorated functional recovery and reduced lesion size in response to injury, by increasing the expression of ERK1/2, brain-derived neurotrophic factor (BDNF), and anti-apoptotic Bcl-2. Similarly, DHA (22:6 n-3) pre-treatment in an acute spinal cord injury model diminished the extent of functional deficits as compared to that observed in the control group, and this protective effect was associated with increased survival of precursor cells, sparing of white matter and axonal preservation (Figueroa et al., 2012; Lim et al., 2013b). In the same way, mice carrying the Fat-1 transgene for boosting endogenous synthesis of n-3 PUFAs showed better outcome after spinal cord injury (Lim et al., 2013a). Finally, in relation to diabetes, it was shown that the augmentation of epoxy-fatty acid resources, as obtained by inhibiting soluble epoxide hydrolase, resulted in a dose dependent anti-allodynic effect on neuropathic pain due to glucose toxicity (Inceoglu et al., 2012).

### FATTY ACIDS AND NEUROLOGICAL DISORDERS

Particular changes in brain fatty acid composition appear to be intimately connected to a series of neurological diseases, as recently reported in several studies. Thus, Conklin et al. (2010) observed a reduction in the quantity of both saturated and unsaturated fatty acids of various types, including n-3 and n-6 PUFAs, in the cingulate cortex of depressive patients. Similar alternations in n-3 PUFAs, including EPA (20:5 n-3) and DHA (22:6 n-3), were also shown by others (Lin et al., 2010). In another study, it was noticed that the altered concentrations of MUFAs and PUFAs were region-specific. In fact, no changes in n-3 and n-6 PUFAs were found in hippocampus and orbitofrontal cortex of patients with depression but concentrations of MUFAs, such as oleic acid (18:1 n-9), and SFAs, such as palmitic acid (16:0), appeared augmented (Hamazaki et al., 2012). A partial confirmation of these findings emerged from another study showing lowered expression of genes involved in PUFA and MUFA synthesis in the frontal cortex of depressed patients (McNamara and Liu, 2011). It is also noteworthy that lifelong n-3 PUFA deficiency perturbed normal endocannabinoid function in prelimbic prefrontal cortex and accumbens, and this effect was related to impaired emotional behavior (Lafourcade et al., 2011). Although less investigated, several studies also detected changes in fatty acids in patients with schizophrenia. A decrease in docosatetraenoic acid (22:4 n-6) was observed in the nuclei of the amygdala of these patients but other PUFAs, including DHA (22:6 n-3) and AA (20:4 n-6), remained unchanged (Hamazaki et al., 2010, 2012). Interestingly, the decrease in total membrane PUFAs found in erythrocytes of young patients with schizophrenia correlated with the degree of demyelination in brain white matter (Peters et al., 2009).

Lastly, several lines of evidence support the anticonvulsant effects of certain fatty acids in animal models of epileptogenesis, and the administration of PUFA enriched diets has been envisaged to treat epileptogenic convulsions. Using the pentylenetetrazol-induced epilepsy rat model, Porta et al. (2009) showed that a PUFA containing diet increased the threshold level for pentylenetetrazol to induce convulsions. A contemporary study confirmed that rats nourished with n-3 PUFAs exhibited greater resistance to pentylenetetrazol-induced seizures (Taha et al., 2009). In the kindling model of epilepsy, intracerebroventricular injection of DHA (22:6 n-3), or its derivative NPD1, limited the progression in the hippocampus of the electrically induced neuronal hyperexcitability characteristic of seizures (Musto et al., 2011). In contrast, other studies did not corroborate these findings, since DHA (22:6 n-3) or EPA (20:5 n-3) showed neither anticonvulsant activity nor protection against pentylenetetrazol-induced seizures (Willis et al., 2009).

## CELLULAR ROLES OF FATTY ACIDS IN THE NERVOUS SYSTEM

### ACTIONS OF FATTY ACIDS IN THE HIPPOCAMPUS

**FIGURE 2:**
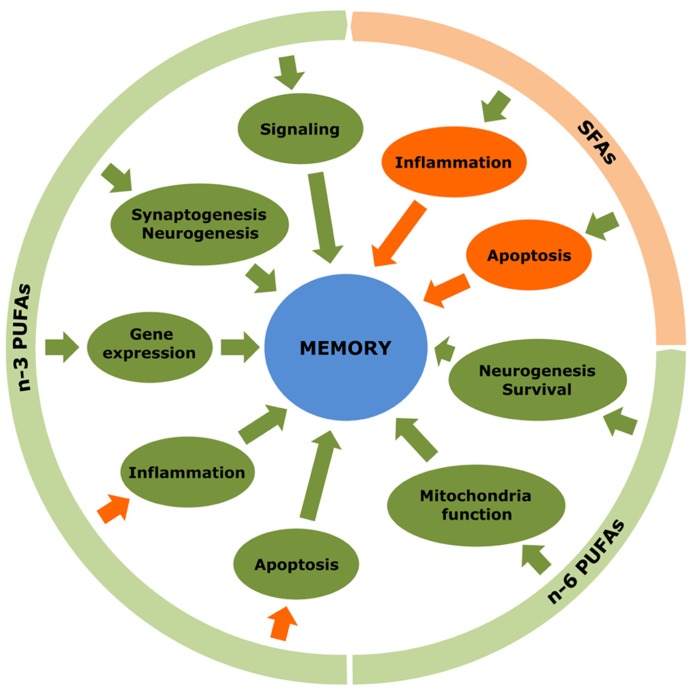
**Multiple effects of fatty acids in the hippocampus.** n-3 and n-6 PUFAs exert a variety of positive actions that promote formation, storage and processing of learning and memory in the hippocampus. In contrast, SFAs display rather negative actions. Green arrows indicate stimulatory effects while orange arrows represent inhibitory effects.

Many recent studies have investigated the implication of fatty acids in learning and memory processes occurring in the hippocampus (**Figure 2**). In general, n-3 PUFAs were shown to foster neuronal activity and hence counteract memory deficits. It is well known that increased c-Fos expression is an indicator of neuronal activity in response to extracellular signals like growth factors, and it is initiated when neurons fire action potentials. Commonly, the activity of c-Fos decreases as age extends and spatial memory goes off. Provision of n-3 PUFAs restored c-Fos expression in the hippocampus, and enhanced neuronal activity ultimately leading to the amelioration of memory deficits in aged mice (Labrousse et al., 2012). Dietary DHA (22:6 n-3) also enhanced the expression of F-ATPase involved in mitochondrial ATP synthesis in the CA1 region of the hippocampus, whereas its deficiency led to decreased glucose transporter expression and defective glucose transport in the cerebral cortex (Harbeby et al., 2012). The stimulatory action of n-3 PUFAs on gene expression also appears to affect neurotransmission. In fact, recent proteomics studies performed on mouse brain deficient in DHA (22:6 n-3) revealed a loss of synaptic proteins associated with altered synaptic transmission (Sidhu et al., 2011). In contrast, expression of vesicular glutamate transporters 1 and 2, which are implicated in glutamatergic neurotransmission, was increased in response to ALA (18:3 n-3) exposure (Blondeau et al., 2009). Similarly, DHA (22:6 n-3) provision to rats with traumatic brain injury enhanced learning ability, by modulating the expression levels of synapsin-1, cAMP response element-binding protein-1 and calcium/calmodulin-dependent protein kinase-2 in the hippocampus of treated animals (Wu et al., 2008, 2011). DHA (22:6 n-3) also ameliorated spatial memory in rats by increasing the expression of subtypes of endocannabinoid/endovanilloid receptors (Pan et al., 2011). Last, n-3 PUFAs augmented the expression of a series of transcription factors involved in learning and memory, including retinoic acid receptor, retinoic X receptor and peroxisome proliferator-activated receptor (Dyall et al., 2010).

Many positive actions of DHA (22:6 n-3), and likely other n-3 PUFAs, may therefore converge to enhance synaptic transmission, and ameliorate spatial learning and memory (Connor et al., 2012). In a mouse model of systemic lupus erythematosus and Sjögren’s syndrome, which is characterized by behavioral abnormalities, reduced aged hippocampal neurogenesis and loss of long-term potentiation (LTP), the dietary supplementation with n-3 PUFAs corrected LTP at synapses in the medial perforant pathway/dentate gyrus and enhanced the amount of adult-born neurons in the hippocampus (Crupi et al., 2012). Similarly, docosapentaenoic acid (DPA, 22:5 n-3) also ameliorated hippocampal function by attenuating the reduction in LTP in aged brain (Kelly et al., 2011). Finally, *in vitro* studies showed that treatment of differentiated PC12 cells with EPA (20:5 n-3) resulted in activation of the neuroprotective PI3-kinase/Akt signaling pathway, a mechanism that might account for the increase in LTP observed *in vivo* following EPA (20:5 n-3) treatment (Wu et al., 2008; Kawashima et al., 2010).

In Alzheimer’s disease, Aβ peptide induces neuronal apoptosis through degradation of the adaptor protein insulin receptor substrate-1 in a c-Jun N-terminal kinase (JNK)-dependent manner. An n-3 PUFA enriched diet prevented the phosphorylation of JNK, and ultimately protected neurons from death *in vitro* and improved cognitive deficit *in vivo* (Ma et al., 2009). Also, lower levels of phosphorylated tau protein and improved brain function were observed by crossing 3xTg-AD mice with Fat-1 expressing mice to enhance the endogenous production of n-3 PUFAs (Lebbadi et al., 2011). Nevertheless, it is noteworthy that 12/15-lipo-oxygenase adversely affected Alzheimer’s disease pathology by synthesizing pro-inflammatory and pro-oxidant hydroperoxyacids resulting from the oxidation of PUFAs, so that genetic ablation of this enzyme ameliorated cognitive function (Yang et al., 2010).

Neuroinflammation is one of the distinctive features of aged or diseased brain, as demonstrated by the activation of glial cells and the increase in the expression of a variety of pro-inflammatory factors. In this respect, it was reported that n-3 PUFA provision restored spatial memory loss in aged animals by suppressing pro-inflammatory interleukin-1β and reverting to normal the morphology of microglia and astrocytes in the hippocampus (Labrousse et al., 2012; Park et al., 2012). n-3 PUFAs also yielded protecting effects to neurons by blocking microglia activation in a transgenic mouse model of systemic lupus erythematosus and Sjögren’s syndrome (Crupi et al., 2012). In the same way, DPA (22:5 n-3) inactivated microglia attenuating neuroinflammation and counteracting spatial learning deficit in aged brain (Kelly et al., 2011). Contrary to the protective effects of PUFAs, SFAs stimulated the secretion of pro-inflammatory cytokines and induced apoptosis in astrocytes. Particularly, palmitic acid (16:0), lauric acid (12:0), and stearic acid (18:0) triggered the secretion of tumor necrosis factor-α (TNF-α) and interleukin-6 by engaging toll-like receptor-4 (TLR-4). Moreover, palmitic acid (16:0) also activated caspase-3 and modified the Bax/Bcl-2 ratio in these glial cells for apoptotic demise. Interestingly, these pro-inflammatory actions of SFAs could be reverted by n-3 PUFAs like DHA (22:6 n-3; Gupta et al., 2012; Wang et al., 2012).

Another way by which n-3 PUFAs can afford neuroprotection is by preventing apoptosis. The mouse model of infantile neuronal ceroid lipofuscinosis, a neurodegenerative disease caused by palmitoyl-protein thioesterase-1 (PPT1) deficiency, manifests enhanced endoplasmic reticulum- and oxidative stress that lead to apoptotic cell demise. In PPT1-deficient cells from such mice, intervention of n-3 PUFAs attenuated stress and repressed apoptotic death casting a protection to neuronal cells (Kim et al., 2010; Wu et al., 2011). Similarly, differentiated PC12 cells treated with EPA (20:5 n-3) showed lower rates of apoptosis and suppressed activity of the apoptotic effector caspase-3 (Boudrault et al., 2009; Kawashima et al., 2010). Conjugated LA (18:2 n-6) also protected neurons from mitochondrial dysfunction and demise. Treatment of cortical neurons with this fatty acid following excitotoxic glutamate exposure resulted in decreased glutamate-induced loss of mitochondrial function, increased Bcl-2 expression and prolonged neuronal survival (Hunt et al., 2010). In the same manner, administration of fish oil, that is a rich source of n-3 PUFAs, protected hippocampal neurons from diabetic insult by precluding the expression of apoptosis inducing genes in both CA1 region and cultured cells, and by increasing the expression of anti-apoptotic genes, such as Bcl-2 and Bcl-xL (Zhang and Bazan, 2010; Zhao et al., 2012). Together with caspase-3, ceramides, resulting from the hydrolysis of sphingomyelin by sphingomyelinase, are well-known apoptosis inducing factors. Treatment with DPA (22:5 n-3) inactivated sphingomyelinase and caspase-3 in the hippocampus of elderly rats (Kelly et al., 2011). On the other hand, n-3 PUFA withdrawal modulated the phosphorylation of glycogen-synthase kinase-3β and ERK1/2, predisposing more hippocampal neurons to damage in an *in vitro* oxygen and glucose deprivation model of ischemia (Moreira et al., 2010b). Along with this, a decrease in the release of PUFAs from cell membranes in the rat hippocampus, as a result of reduced phospholipase-A2 activity, caused alterations in membrane fluidity that could account for loss of spatial memory and cognitive impairment in Alzheimer’s disease (Schaeffer et al., 2011). However, the protective effects of n-3 PUFAs under certain conditions seemed to be limited to some of the members of this class of fatty acids. Thus, only DHA (22:6 n-3) offset the expression of AMPA receptors in the membrane of hippocampal neurons and attenuated neurotoxicity leading to improved cognitive function. Other members of the n-3 PUFA family, especially EPA (20:5 n-3), lacked such a protective effect against AMPA-mediated toxicity (Ménard et al., 2009).

Synaptogenesis is one of the mechanisms by which memory process takes place. Hence, the loss of synapses is characteristic of neurodegenerative conditions and aging. For instance, cortical or hippocampal neurons incubated with the neurotoxic prion-derived peptide PrP82–146, and pre-treated with DHA (22:6 n-3) or EPA (20:5 n-3), showed less loss of synaptophysin-1 and reduced accumulation of prion peptide (Bate et al., 2010). ALA (18:3 n-3) also stimulated the expression of genes involved in synaptic function, like VAMP-2, SNAP-25 and synaptophysin-1, that led to improved stability and physiology of synapses (Blondeau et al., 2009). Similarly, the chronic supplementation of n-3 PUFAs yielded anti-depressant effects by increasing the expression of synaptophysin-1 in the hippocampus (Venna et al., 2009). However, another study performed on SH-SY5Y cells reported that DHA (22:6 n-3) did not affect the neurotransmission machinery, as evaluated by the expression of synaptotagmin-1, syntaxin-1A, and synaptobrevin-1, although the release of noradrenaline by these cells was enhanced (Mathieu et al., 2010).

Hippocampal neurogenesis also contributes to learning and memory processes. The mouse model of systemic lupus erythematosus and Sjögren’s syndrome typically exhibits age-dependent reduced hippocampal neurogenesis. Supplementation of diet with n-3 PUFAs to these mice enhanced the density of bromodeoxyuridine (BrdU)- and doublecortin positive cells in the hippocampus, suggesting an ongoing neurogenesis (Crupi et al., 2012). Similar neurogenesis enhancement was also reported in response to ALA (18:3 n-3) treatment (Blondeau et al., 2009). In addition, AA (20:4 n-6) even increased neurogenesis at postnatal stages when administered at gestation period (Maekawa et al., 2009). Several *in vitro* studies revealed that not only n-3 PUFA precursors, such as EPA (20:5 n-3), but also naturally derived metabolites, including the neurotrophic N-docosahexaenoylethanolamine, stimulated neurogenic differentiation of neural stem cells (Katakura et al., 2013; Rashid et al., 2013). The importance of the stimulatory role of PUFAs for neurogenesis is also illustrated by experiments reporting increased expression of fatty acid binding proteins (FABPs) in the ischemic hippocampus. FABPs are carriers of PUFAs in the cytoplasm, and their expression declines with age in association with reduced synaptic activity and other cellular functions. CA1 and dentate gyrus regions in the hippocampus showed augmented levels of FABP-5 and FABP-7 after ischemia, suggesting elevated transportation of PUFAs in these regions to restore cellular neurophysiology (Liu et al., 2010; Ma et al., 2010). More importantly, at post-ischemic stages, the subgranular zone in the dentate gyrus of the hippocampus, a niche of adult neurogenesis, displayed a concomitant increase in the neuronal expression of FABPs and the fatty acid receptor GPR40, representing compensatory processes of newborn cells (Boneva et al., 2011a,b; Yamashima, 2012). Finally, it is noteworthy that many of the beneficial actions of PUFAs on hippocampal function were associated with an increase in the production of BDNF, which is a member of the neurotrophin family of growth factors involved in supporting growth, differentiation and survival of neurons (Wu et al., 2008, 2011; Blondeau et al., 2009; Venna et al., 2009; Avraham et al., 2011; Vines et al., 2012).

### ACTIONS OF FATTY ACIDS IN THE HYPOTHALAMUS

The central regulation of energy balance involves a number of neuronal circuits in the hypothalamus that either exert anorexic actions or stimulate food intake. In this respect, it was recently shown that certain fatty acids could influence the control of energy homeostasis by the hypothalamus. In general, dietary supplementation with fish oil, rich in n-3 PUFAs, normalized several hypothalamic neurochemical systems in food restricted animals (Avraham et al., 2011). However, supplementation of diet with SFAs induced endoplasmic reticulum stress and expression of cytokines via TLR-4 signaling in the hypothalamus, and this effect resulted in resistance to anorexigenic signals (Milanski et al., 2009). At the cellular level, treating hypothalamic mHy-poE-44 cells with palmitic acid (16:0) increased the expression of the orexigenic neuropeptide-Y, suggesting that this fatty acid could enhance food intake (Fick et al., 2011). Moreover, palmitic acid (16:0) faded insulin signaling and enhanced endoplasmic reticulum stress and caspase-3 cleavage in the same cell line, which resulted in apoptosis in a JNK-dependent manner (Mayer and Belsham, 2010). In another study, exposure to palmitic acid (16:0) displayed no effects on insulin resistance and inflammatory process activation but corroborated the stimulation of endoplasmic reticulum stress and apoptosis, along with the activation of mitogen-activated protein kinase (Choi et al., 2010).

### ACTIONS OF FATTY ACIDS IN THE NIGROSTRIATAL PATHWAY

**FIGURE 3:**
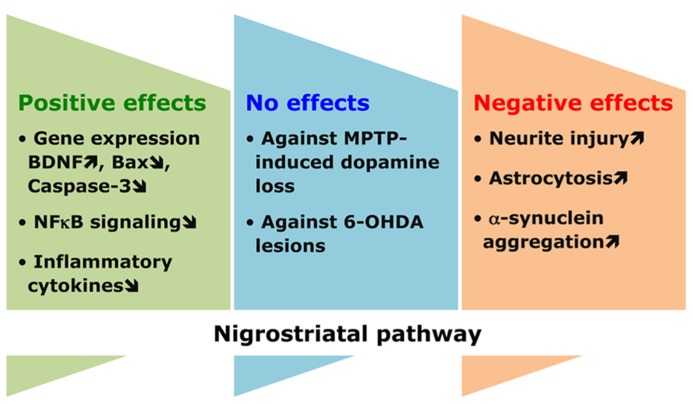
**Conflicting effects of n-3 PUFAs in the nigrostriatal pathway.** n-3 PUFAs are commonly endowed with a wide range of helpful effects, as illustrated by the protective benefit that these fatty acids offer to dopaminergic neurons in the nigrostriatal tract against apoptotic and pro-inflammatory cues. However, extreme caution should be exercised since these same PUFAs may not provide complete safety to halt degeneration induced by parkinsonian toxins or even trigger adverse effects, which eventually aggravates the extent of the pathological process.

Growing evidence supports a link between the dietary intake of n-3 PUFAs and the function (or dysfunction) of the nigrostriatal pathway involved in the control of movement (**Figure 3**). This relationship was particularly investigated in a number of animal models of Parkinson disease, which is a neurodegenerative condition primarily characterized by the loss of dopaminergic neurons connecting the substantia nigra to the striatum. In several recent studies, n-3 PUFAs were shown to be beneficial by reverting disease phenotype. In the MPTP model of Parkinson disease, pre-treatment of mice with n-3 PUFAs bestowed protection by increasing the expression of BDNF and involving its TrkB receptor (Bousquet et al., 2009; Balanzá-Martïnez et al., 2011). In other studies, it was found that exposure to the n-3 PUFA ethyl-eicosapentaenoate derivative lowered the expression of Bax and caspase-3, and enhanced cortical dopamine levels (Bousquet et al., 2008; Meng et al., 2010). Furthermore, n-3 PUFAs also yielded protective influence indirectly, by attenuating inflammation-causing factors. These fatty acids targeted the NFκB signaling pathway in microglia to suppress their over-activated response and hence protect dopaminergic neurons (Boudrault et al., 2009; Zhang et al., 2010; Ji et al., 2012; Zhou et al., 2012).

Other findings, however, did not support the beneficial effects of n-3 PUFAs on Parkinson disease. It was reported that treatment with ethyl-eicosapentaenoate, although minimized pro-inflammatory cytokines and yielded positive effects on procedural memory deficit, it was unable to preclude the loss of nigrostriatal dopamine in MPTP mice (Shchepinov et al., 2011; Luchtman et al., 2012). Similarly, the parkinsonian neurotoxin 6-hydroxydopamine caused lesions in the medial forebrain bundle of rats and motor deficits that remained unaffected by fish oil derived n-3 PUFAs (Delattre et al., 2010). A chronic intervention of a DHA (22:6 n-3) containing diet modified neither the number of cortical glial cells nor the expression of α-synuclein, which is typically involved in disease pathogenesis (Muntané et al., 2010). The use of different animal models of Parkinson disease and the different ways of treating these mice to counteract the pathological process may explain the observed discrepancies. In this respect, it is important to mention that some studies indicated even adverse effects of n-3 PUFAs on Parkinson disease pathogenesis. Indeed, the presence of DHA (22:6 n-3) augmented neuritic injury and astrocytosis in mice transgenic for a Parkinson disease causing mutation in human α-synuclein. In addition, DHA (22:6 n-3) triggered oligomerization of α-synuclein, through the activation of retinoic X receptor and peroxisome proliferator-activated receptor-γ2. Interestingly, its withdrawal from diet was found to be beneficial against the deleterious effects caused by it provision (Yakunin et al., 2012). Finally, structural and conformational modifications in α-synuclein leading to pathological aggregation were brought by DHA (22:6 n-3; De Franceschi et al., 2009, 2011; Bousquet et al., 2011).

### ACTIONS OF FATTY ACIDS IN THE PERIPHERAL NERVES

A subset of peripheral sensory neurons expresses transient receptor potential cation channel-A1 (TRPA1), which is involved in pain and neurogenic inflammation. TRPA1 is a target for a variety of noxious and inflammatory irritant substances. In addition, it was found that n-3 PUFAs could act as a ligand for TRPA1 to excite sensory neurons and hence regulate their responses *in vivo* (Motter and Ahern, 2012). Transient receptor potential vanilloid cation channel-1 (TRPV1), which is another member of the family, is also found mainly in nociceptive neurons of the peripheral nervous system, where they are involved in transmission and modulation of pain. In this respect, it was shown that NPD1, which has anti-inflammatory properties, inhibited TRPV1 currents induced by capsaicin in dorsal root ganglion neurons, and modulated TRPV1/TNF-α-mediated synaptic plasticity in the spinal cord, suggesting a novel analgesic role (Park et al., 2011). The effects of fatty acids on sensory neurons go beyond receptor signaling. Both n-6 and n-3 PUFAs promoted neurite outgrowth in sensory neurons from dorsal root ganglia of embryos but also adult and aged animals (Robson et al., 2010). Enhanced levels of endogenously synthesized n-3 PUFAs also bestowed beneficial effects in various aspects. Thus, dorsal root ganglion neurons from Fat-1 expressing mice exhibited more resistance to hypoxia and mechanical injury as compared to neurons from wild-type littermates. Furthermore, Fat-1 expressing mice showed better functional recovery after sciatic nerve crush. The increased endogenous levels of n-3 PUFAs reduced the expression of the stress sensor activating transcription factor-3 in dorsal root ganglion neurons, and diminished muscle atrophy (Gladman et al., 2012). Similarly, our own studies also reported that the down-regulation of SCD1, which is in charge of the production of MUFAs such as oleic acid (18:1), triggered accelerated motor function recovery after sciatic nerve crush, providing evidence for a new role of this fatty acid desaturase in modulating the restorative potential of the neuromuscular axis (Hussain et al., 2013).

The retina possesses a high concentration of n-3 PUFAs, particularly DHA (22:6 n-3). Many studies have shown that this fatty acid not only has a structural function but also protects visual neurons from trauma and disease. Recently, it was noticed that the retinal dysfunction induced by diabetes could be recovered to some extent by supplementing DHA (22:6 n-3) extraneously. In fact, diabetes resulted in reduced levels of n-3 PUFAs, by affecting n-3 fatty acid desaturase enzymatic activity, so that the provision of a DHA (22:6 n-3) enriched diet prevented dysfunction of rods and ameliorated vision (Yee et al., 2010). Also, n-3 PUFA derived NPD1, together with pigment epithelial-derived growth factor, promoted corneal nerve regeneration in a rat model of surgical injury (Cortina et al., 2010, 2012; Kenchegowda et al., 2013). However, other studies rather obtained contradictory results. Therefore, augmented levels of DHA (22:6 n-3) bestowed no protection against retinal degeneration in mice carrying a disease-causing VPP rhodopsin mutation and expressing Fat-1 (Li et al., 2009, 2010). In the same way, it was also reported that high levels of DHA (22:6 n-3) in the retina could generate oxidative stress, instead of protection, and hence enhance the susceptibility to degeneration (Tanito et al., 2009).

## CONCLUSION

The biological functions of fatty acids have been investigated intensively during these last years, due to their active involvement in the physiology of both central and peripheral nervous system. They promote brain development, ameliorate cognitive functions in normal and diseased conditions, serve as anti-depressants and anti-convulsants, bestow protection against traumatic insults, and elevate repairing processes. At the cellular level, fatty acids stimulate gene expression and neuronal activity, and boost synaptogenesis and neurogenesis while preventing from neuroinflammatory toxicity and apoptosis (**Figure 2**). Although the demand for fatty acids in a healthy body applies to any of them, it can be said that, in general, excess of SFAs and, to some extent, n-6 PUFAs brings about negative consequences, whereas MUFAs and n-3 PUFAs are endowed with rather beneficial properties. In this respect, the ratio of n-6 to n-3 PUFAs is of special interest. It has been postulated that a relatively constant n-6:n-3 ratio of about 1:1 constituted a major breakthrough in the expansion of gray matter in the cerebral cortex of modern human beings (Bradbury, 2011). In the brain, the preservation of an optimal n-6:n-3 ratio is crucial to the maintenance of the variety of the cellular processes in which PUFAs participate (Luchtman and Song, 2013). During the last century, however, the n-6:n-3 ratio has dramatically increased up to 20–25:1, particularly in Western societies, because of a high consumption of n-6 PUFAs to the detriment of n-3 PUFA intake (Simopoulos, 2011). Once the equilibrium is broken, an excessively high n-6:n-3 ratio would impair normal brain function and, importantly, predispose to disease (Palacios-Pelaez et al., 2010). According to what we have exposed herein, a huge amount of studies have shown the good and the bad side of different fatty acids in many experimental models of trauma and disease. Nevertheless, the diversity in modeling any given physiopathological condition, together with differences in time, dose and type of fatty acid used to counteract the insult, certainly account for a number of conflicting results concerning the nature of the observed effects. In addition, it must be taken into consideration that particular fatty acids are assumed to foster neuroprotection but engender indeed a series of collateral deleterious actions, such as increasing oxidative stress susceptibility or favoring neurodegenerative protein aggregation, which may preclude the use of these fatty acids under certain (pathological) conditions (**Figure 3**). Finally, it is also noteworthy that, frequently, studies used nutritional approaches consisting in giving a specific fatty acid or its precursor mixed with others and forming part of foods relatively more complex than desired, since they also contain other substances with potential, uncontrolled positive or negative effects. Taken together, these drawbacks limit the translatability of successful results in terms of neuroprotection obtained in animal experiments into effective therapeutic interventions in humans. Numerous epidemiological studies have put fatty acids forward as key factors contributing to neuropathology but, in some cases, discrepant concentrations of fatty acids were reported in the corresponding diseased brain regions (**Table 2**). Despite these constraints, on the basis of these epidemiological studies and supported by experimental research, there is quite realistic evidence to envisage that nutritional therapies based on fatty acids can be of benefit to several neurodegenerative and neurological diseases, such as age-related macular degeneration, cognitive decline, depression, and some related behavioral disorders (Prior and Galduróz, 2012; Schleicher et al., 2013). More research is needed now for arriving at the final and conclusive result concerning the type of fatty acid, number of double bonds, origin, particular stage and proper concentration to achieve beneficial therapeutic potential against otherwise incurable diseases.

## Conflict of Interest Statement

The authors declare that the research was conducted in the absence of any commercial or financial relationships that could be construed as a potential conflict of interest.
